# Nucleoporin 98 mislocalization is a common feature in primary tauopathies

**DOI:** 10.1093/braincomms/fcad097

**Published:** 2023-03-28

**Authors:** Niharika Nag, Timir Tripathi

**Affiliations:** Molecular and Structural Biophysics Laboratory, Department of Biochemistry, North-Eastern Hill University, Shillong 793022, India; Molecular and Structural Biophysics Laboratory, Department of Biochemistry, North-Eastern Hill University, Shillong 793022, India; Regional Director’s Office, Indira Gandhi National Open University (IGNOU), Regional Centre Kohima, Kohima 797001, India

## Abstract

This scientific commentary refers to ‘Altered localization of nucleoporin 98 in primary tauopathies’ by Dickson *et al*. (https://doi.org/10.1093/braincomms/fcac334).

**This scientific commentary refers to ‘Altered localization of nucleoporin 98 in primary tauopathies’ by Dickson *et al*. (https://doi.org/10.1093/braincomms/fcac334).**

Tauopathies include a range of neurodegenerative diseases characterized by an accumulation of the microtubule-associated protein tau (MAPT). Tau, a natively unfolded protein, can aggregate to form paired helical filaments (PHFs) and neurofibrillary tangles (NFTs) in several tauopathies, including Alzheimer’s disease, progressive supranuclear palsy (PSP), Pick disease, Huntington disease, frontotemporal lobar degeneration-tau (FTLD-tau), corticobasal degeneration (CBD), etc. A common feature recognized in several neurodegenerative disorders has been the disruption of the nuclear pore complex (NPC) and the nucleocytoplasmic transport (NCT).^1,2^ The NPC is a large protein complex lodged in the nuclear envelope and composed of around 30 different proteins called nucleoporins (Nups). The Nup proteins make up the three main structural components of the NPC, namely, the cytoplasmic filaments, a central pore and a nuclear basket. The proteins lining the central pore are called the FG-Nups, which are disordered proteins comprising phenylalanine–glycine repeat motifs. The FG-Nups are crucial in forming the selectively permeable barrier of the NPC, which regulates the NCT of biomolecules. One of the essential proteins forming the barrier is Nup98.^3^ It has been shown that Nup98 interacts with tau and is mislocalized into the cytoplasm in Alzheimer’s disease.^4^ In their recent article in *Brain Communications*, Dickson *et al.* reported that Nup98 is mislocalized in the frontal cortex of several primary tauopathies like FTLD-tau, CBD and PSP.^5^ The data indicate that Nup98 mislocalization is a general feature of primary tauopathies and is associated with pathological tau accumulation and neurotoxicity (Fig. 1).

**Figure 1:**
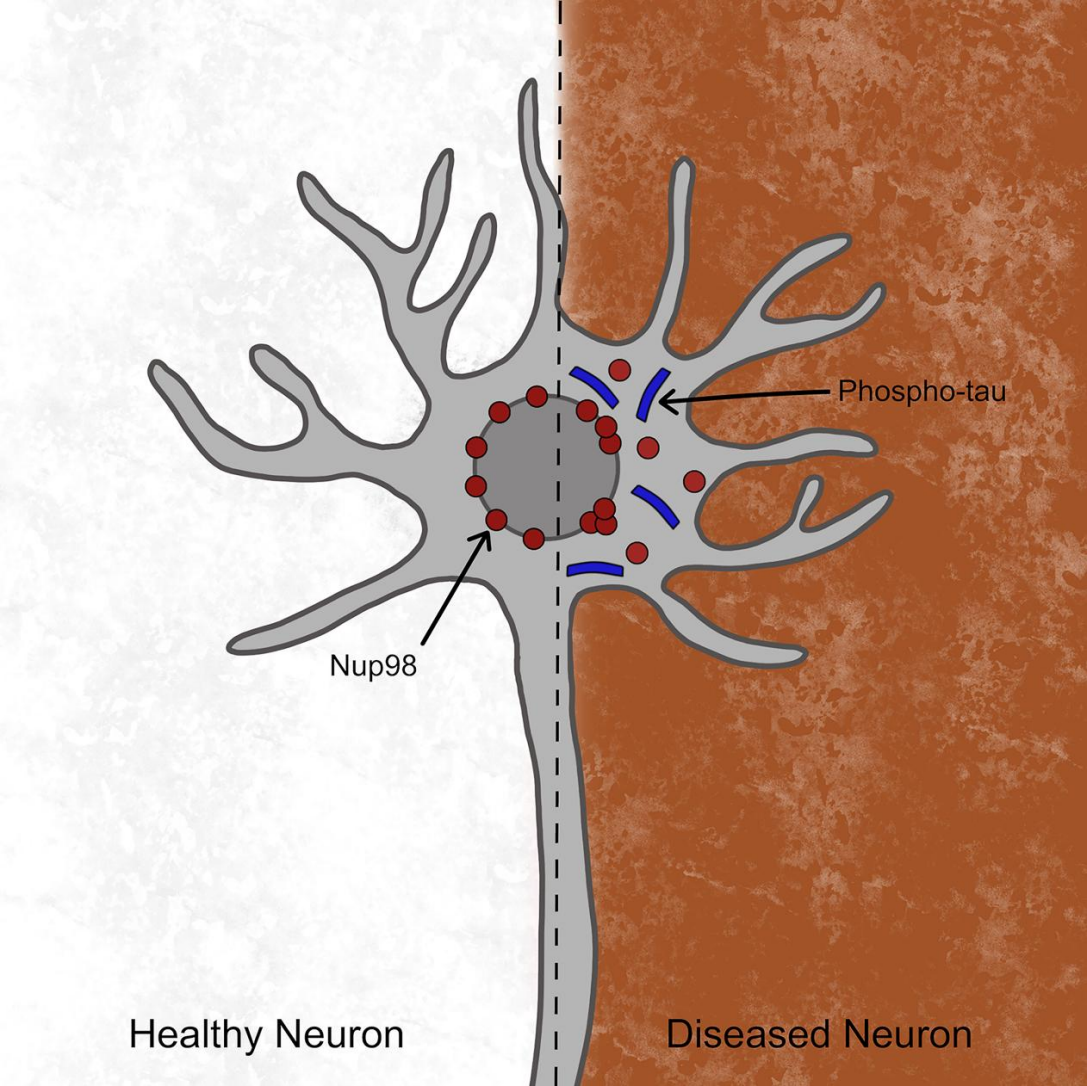
**Schematic diagram of a healthy neuron (left side) versus a diseased neuron (right side) in primary tauopathies.** In the healthy neuron, in the absence of phospho-tau, there is no mislocalization of Nup98. On the other hand, in the diseased neuron of primary tauopathies, there is the presence of phospho-tau and nuclear and cytoplasmic mislocalization of Nup98.

The authors used confocal microscopy to examine the staining pattern of Nup98 in the frontal cortex tissue of the control and those from FTLD-tau, CBD and PSP. They observed that though the neurons of the control frontal cortex showed a uniform distribution of Nup98 on the nuclear membrane, the neurons of the primary tauopathy tissues showed aberrant localization of Nup98. The mislocalized Nup98 was found non-uniformly distributed on the nuclear membrane and in the cytoplasm. Using virtual slide images, the number of neurons containing mislocalized Nup98 and AT8-positive (monoclonal antibody for Ser202, Thr205 phospho-tau) phospho-tau were quantified. The frontal cortex for FTLD-tau, CBD and PSP had a significantly higher percentage of neurons with aberrant nuclear localization of Nup98 than the control. In the case of cytoplasmic mislocalization of Nup98, the percentage of abnormal neurons was higher in the frontal cortex tissue of FTLD-tau and CBD than in control. There was also an increased percentage of neurons with cytoplasmic mislocalization of Nup98 in PSP cases, but the numbers were not statistically significant. When a correlation analysis of the mislocalized Nup98 with AT8 staining was conducted, it was found that both the nuclear and cytoplasmic mislocalized Nup98 were significantly correlated to the number of AT8-positive neurons, with *P* = 0.0062 and *P* = 0.0076, respectively.

The authors then conducted immunohistochemical staining to assess whether mislocalized Nup98 were present in all neurons in the diseased state or if some areas were spared pathologically with no mislocalization of the protein. The confocal microscopy images revealed the primary visual cortex as normal uniformly stained nuclear envelopes with no presence of Nup98 in the cytoplasm in both the control as well as in the cases of the three primary tauopathies. Additionally, there was largely no AT8 staining in the neurons of the primary visual cortex, depicting the absence of phospho-tau in the tissues. When quantified, there was no significant difference in the abnormal nuclear and cytoplasmic mislocalization of Nup98 between the cases of primary tauopathies and the control. An important observation here was that the cases with the highest percentage of Nup98 mislocalization were seen in the two cases of FTLD-tau, having the P301L mutation in tau, which is one of the most common types of pathogenic mutations of tau.

There have been several observations of impaired NCT and structural aberrations in NPCs in Alzheimer’s disease,^6^ followed by the discovery of the direct interaction of the proteins of the NPC with tau, which led to the mislocalization of Nups. Phosphorylated tau was found to interact with Nup98, leading to its mislocalization.^4^ In the current study, the authors discovered that this phenomenon is not confined only to Alzheimer’s disease but is also prevalent in other primary tauopathies like FTLD-tau, CBD and PSP. Though the current study does not look into the mechanism behind the Nup98 mislocalization, a direct interaction of pathological phospho-tau and Nup98 could be the cause of mislocalization of Nup98 in these tauopathies based on the fact that there exists a correlation between the number of AT8-positive neurons and neurons with abnormal nuclear and cytoplasmic localization of Nup98, combined with the evidence of interaction between Nup98 and tau in Alzheimer’s disease.^7^ The mislocalization of Nup98 suggests that the NCT in these tauopathies is compromised. The disruption of the NCT caused by an interaction between phospho-tau and Nup98, a common feature in tauopathies, provides an opportunity to perhaps come up with a common therapeutic strategy to combat diverse primary neuropathies. This opens up avenues for the design and development of drugs that can target the interaction between the proteins to counteract the disruption of the NCT. There is also evidence that the removal of phospho-tau can reverse the mislocalization of Nup98.^4^ Perhaps by reducing or targeting the hyper-phosphorylation of tau, NPC and NCT health may be restored. The disruption of the NCT in neurons is a phenomenon that has also been observed in many other neurodegenerative diseases like Parkinson’s disease, Huntington’s disease, amyotrophic lateral sclerosis (ALS), etc. Other proteins involved in NCT, like the FG-Nup Nup62, and nuclear transport receptors (NTRs), like importin-α5 and β1, have also been found to be compromised in neurodegenerative diseases. Nup62, a crucial component of the NPC permeability barrier, has been found to be involved in several neurodegenerative diseases, including Alzheimer’s disease, Huntington’s disease and ALS/FTLD.^8,9^ In ALS/FTLD, the Nup62 form condensates with the protein TDP-43. Condensates formed through the process of phase separation have been known to be involved in neurodegenerative diseases by being a precursor to pathological aggregates.^10^ Understanding the role of these other proteins and their interactions in tauopathies would further aid in getting a comprehensive knowledge of the underlying mechanisms of the diseases and, therefore, in producing therapeutic approaches against them.
